# Moxibustion exhibits therapeutic effects on spinal cord injury via modulating microbiota dysbiosis and macrophage polarization

**DOI:** 10.18632/aging.204184

**Published:** 2022-07-21

**Authors:** Zhuang Zhang, Rubo Sui, Lili Ge, Dongjian Xia

**Affiliations:** 1 Department of Neurology, The First Affiliated Hospital, Jinzhou Medical University, Jinzhou, Liaoning 121012, China; 2 Department of Ultrasound, The First Affiliated Hospital, Jinzhou Medical University, Jinzhou, Liaoning 121012, China; 3 Department of Neurosurgery, The First Affiliated Hospital, Jinzhou Medical University, Jinzhou, Liaoning 121012, China

**Keywords:** spinal cord injury, moxibustion, macrophage polarization, microbiota dysbiosis, inflammation

## Abstract

In this study, we aimed to study the effect of moxibustion (MOX) on microbiota dysbiosis and macrophage polarization, so as to unveil the mechanism underlying the therapeutic effect of MOX in the management of spinal cord injury (SCI). SCI animal models were established to study the effect of MOX. Accordingly, it was found that MOX treatment significantly suppressed the Ace index and Shannon index in the SCI group. Moreover, the reduced relative levels of Lactobacillales and Bifidobacteriales and the elevated relative level of Clostridiales in the SCI animals were mitigated by the treatment of MOX. The body weight, food intake, energy expenditure (EE) index and respiratory quotient (RQ) index of SCI mice were all evidently decreased, but the levels of interleukin (IL)-17, interferon (IFN)-γ, monocyte chemoattractant protein-1 (MCP-1) and IL-1β were increased in the SCI group. Moreover, MOX treatment significantly mitigated the dysregulation of above factors in SCI mice. Accordingly, we found that the Basso Mouse Scale (BMS) score was negatively correlated with the level of Clostridiales while positively correlated with the level of Lactobacillales. The apoptotic index and caspase-3 level were both evidently increased in the SCI group, while the SCI+MOX group showed reduced levels of apoptotic index and caspase-3. Therefore, it can be concluded that the treatment with MOX can promote microbiota dysbiosis and macrophage polarization, thus alleviating spinal cord injury by down-regulating the expression of inflammatory cytokines.

## INTRODUCTION

After the onset of spinal cord injury (SCI), the reduction of control over sympathetic nerve cells renders the autonomic reflex circuits in the nervous system dysfunctional, triggering pathology such as dysreflexia as well as SCI [1–3]. Such disorders induce the imbalance in the autonomic signals in the digestive tract, leading to dysfunctions in mucosal secretion, colonic mobility, as well as vascular tone [4, 5]. Amongst the problems faced by people with SCI, neurogenic bowel dysfunction (NBD) has actually been a significant issue that may seriously impact the life of SCI patients. Two major indications of NBD, i.e., constipation as well as fecal incontinence, occur in nearly half of SCI patients [6, 7].

Moxibustion has been a treatment used in traditional Chinese medicine for many years to induce pain relief at the points of acupuncture called acupoints [8]. Moxibustion trigger self-healing by impacting the functions of the neuroendocrine immune system [9]. While the use of thermal therapy to replace moxibustion has been controversial, both treatments involve similar mechanisms in pain relief as well as self-healing [9, 10]. Past research actually presented that moxibustion modifies the expression levels of genes such as cytokines to play a beneficial role in the treatments of inflammatory as well as autoimmune conditions [11]. Moreover, it has been found that the combined treatment of moxibustion with acupuncture could recover the motor function and preserve the neuron cells in SCI rats [12].

It was discovered that moxibustion therapy elevated the levels of Firmicutes while reducing the number of Bacteroidetes as well as Proteobacteria. Regarding the efficacy of moxibustion therapy, it was proposed that short term moxibustion therapy might dramatically impact the microbiome in the digestive tract. In general, in ulcer colitis mice, the quantity of beneficial bacteria was reduced, while the number of numerous opportunistic pathogens was increased [13].

Discrepancies of microbiota composition in the gut are called “dysbiosis” and are actually triggered by a lot of factors, such as genes in the host, lifestyle, as well as direct exposure to microbes or even different clinical procedures [14]. Dysbiosis has been linked to both inflammation in the intestinal tract irritation as well as many health conditions outside the digestive tract, like obesity, atopic eczema, allergic reaction, as well as diabetic issues [3, 15, 16].

As reported by previous studies, the severe neurological and psychological complications induced by SCI could induce gut dysbiosis. For example, SCI patients could be subjected to sudden and dramatic life changes, causing acute and chronic psychological stress. Neurogenic damages could develop in bladder and bowel due to spinal autonomic circuitry after SCI. And dysautonomia induced by SCI could also damage the immune system, increasing the patients’ possibility to use antibiotics against infections. Therefore, it was presented that SCI induces gut dysbiosis, which in turn hinders the healing as well as worsening the pathology of SCI [17]. In addition, microbiota in the digestive tract can impact serotonin formation, neurotransmission, as well as metabolic process in a sex-specific way [18, 19].

Macrophage polarization appears to induce different macrophage reactions depending on the perceived stimulations. Macrophage polarization has actually been characterized into 2 types, i.e., M1 as well as M2 polarization, with each type of polarization pertaining to particular immune system feedbacks, amongst which both the advancement as well as relief of inflammation play crucial roles [20].

It has been shown that gut dysbiosis could exacerbate neurological impairment and spinal cord pathology during post-SCI recovery, and hence was considered as a disease-modifying factor after traumatic spinal cord injury [17, 21]. In this study, we aimed to study the effect of MOX on microbiota dysbiosis and macrophage polarization to unveil the mechanism underlying the therapeutic effect of MOX on SCI treatment. Accordingly, we hypothesized that MOX could alleviate spinal cord injury by promoting microbiota dysbiosis and inhibiting the polarization of macrophages.

## MATERIALS AND METHODS

### Animal model

The experimental processes of animal studies have actually been reviewed and approved by our Animal Ethics Committee. Female C57BL/6 adult mice weighing 20 to 22 g were purchased from the Facility of Experimental Animals at our institution and housed in an air-conditioned animal facility subjected to a 12 h: 12 h light:dark cycle. The temperature in the animal facility was maintained at 22 ± 1°C while the humidity level in the animal facility was kept to 50 ± 10%. All mice had *ad libitum* access to food as well as water during the study. Subsequently, the mice were divided into 4 groups, i.e., 1. SHAM group (*N* = 8, mice treated with a sham surgical procedure); 2. SHAM +MOX group (*N* = 8, mice treated with a sham surgical procedure and then given MOX); 3. SCI group (*N* = 8, mice treated with a surgical procedure to induce SCI); and 4. SCI + MOX group (*N* = 8, mice treated with a surgical procedure to induce SCI and then given MOX). To induce SCI, the mice in SCI groups were anesthetized by using 2% isoflurane. Then, laminectomy was conducted at the level of T10 and the mice got a contusion injury induced by dropping an impactor (Infinite Horizons, Precision Systems and Instrumentation, Lexington, KY, USA). After that, the laceration was sutured and the mice were returned to a warming enclosure till they were fully awake. Post-operatively, the animals were given 0.5 ml of Ringer's solution via S.C. injection once a day for 5 times. The mice in the SHAM groups were given laminectomy at level T10 without receiving the injury induced by the impactor. And for the MOX treatment, moxibustion was delivered by using moxa cones that were 0.6 centimeter tall, 0.5 centimeter in diameter and 1.8 gram in weight (Nanyang Hanyi Moxa, Nanyang, China). The moxa cones were positioned on bilateral acupoints ST25 (acupoints as illustrated in Supplementary Figure 1) to deliver 10 minutes of moxibustion once daily for 28 days. The skin temperature at the site of acupuncture was kept at 43°C during the entire treatment procedure. By the completion of this study, all mice were sacrificed by a lethal intravenous dose of sodium pentobarbital at 100 mg/kg.

### RNA isolation and real time PCR

A Trizol reagent (Invitrogen, Carlsbad, CA, USA) was utilized in accordance with the recommended protocol provided by the manufacturer to isolate total RNA content from cell as well as tissue samples. The concentration as well as quality of isolate total RNA samples was identified by utilizing a Nano Drop spectrophotometer (Thermo Fisher Scientific, Waltham, MA, USA) in accordance with the recommended protocol provided by the manufacturer. Then, reverse transcription of isolate total RNA was done by using a Hairpin-it real time PCR Quantitation assay kit (GenePharma, Shanghai, China) along with a PrimeScript RT Master Mix (TaKaRa, Tokyo, Japan) in accordance with the recommended protocol provided by the assay kit manufacturers. In the next step, real time PCR was executed by using a SYBR Green PCR master mix (Applied Biosystems, Foster City, CA, USA) on an 7900HT real time PCR machine (Applied Biosystems, Foster City, CA, USA) in accordance with the recommended protocol provided by the manufacturers. Finally, the relative expression of M2 markers of colonic macrophages, i.e., CD206 (Forward primer sequence: 5′-AGCCAACACCAGCTCCTCAAGA-3′; Reverse primer sequence: 5′-CAAAACGCTCGCGCATTGTCCA-3′) and Arg-1 (Forward primer sequence: 5′-TCATCTGGGTGGATGCTCACAC-3′; Reverse primer sequence: 5′-GAGAATCCTGGCACATCGGGAA-3′), as well as M1 markers of colonic macrophages, i.e., CXCL-9 (Forward primer sequence: 5′-CTGTTCCTGCATCAGCACCAAC-3′; Reverse primer sequence: 5′-TGAACTCCATTCTTCAGTGTAGCA-3′), iNOS (Forward primer sequence: 5′-GCTCTACACCTCCAATGTGACC-3′; Reverse primer sequence: 5′-CTGCCGAGATTTGAGCCTCATG-3′) and caspase-3 (Forward primer sequence: 5′-GGAAGCGAATCAATGGACTCTGG-3′; Reverse primer sequence: 5′-GCATCGACATCTGTACCAGACC-3′), was calculated by normalization to the expression of internal control gene GAPDH (Forward primer sequence: 5′-GTCTCCTCTGACTTCAACAGCG-3′; Reverse primer sequence: 5′-ACCACCCTGTTGCTGTAGCCAA-3′) via making use of the 2^−ΔΔCT^ procedure.

### Western blot analysis

Total protein was separated from tissue samples and assessed by utilizing a BCA protein assay kit (Tiangen Biotech, Beijing, China) in accordance with the recommended protocol provided by the manufacturer. Then, 10 μg of proteins in every sample were resolved by using 10% SDS-PAGE before they were electro-transferred onto polyvinylidene fluoride (PVDF) membranes (Millipore, Billerica, MA, USA) for immunoblotting. Then, the membranes were blocked with 10 FBS and incubated with anti-caspase-3 primary antibodies (dilution of 1:5000; ab32351, Abcam, Cambridge, MA, USA) as well as HRP-labeled secondary antibodies (dilution of 1:1000; ab6747, Abcam, Cambridge, MA, USA) in accordance with the recommended protocol provided by the antibody manufacturer. Finally, the grey levels of protein bands were quantified by utilizing Image J software to determine the protein expression of caspase-3 in each sample.

### ELISA

After the behavior examinations, all mice were killed through cervical dislocation and their blood samples were collected. In the next step, the expression of IL-17, IFN-γ, MCP-1, and IL-1β in each sample was determined by utilizing commercial ELISA kits (Catalog # BMS6001 for IL-17; Catalog # KMC4021 for IFN-γ; Catalog # EMCCL12 for MCP-1; Catalog # BMS6002 for IL-1β; Thermo Fisher Scientific, Waltham, MA, USA) in accordance with the recommended protocol provided by the kit manufacturer.

### TUNEL assay

The status of apoptosis in collected tissue samples was evaluated by using a TUNEL assay (Roche, Basel, Switzerland) in accordance with the recommended protocol provided by the assay kit manufacturer.

### BMS score

The Basso Mouse Scale (BMS) score was assessed based on hindlimb movement after the moxibustion treatment. In brief, the mice were analyzed in an open area for 4 minutes pre-operatively as well as on D3, D7, D14, D21 and D28 postoperatively. The left and right hindlimb performance was ranked independently before the scores averaged to produce the total score and sub-scores of BMS. Locomotion parameters were evaluated by utilizing DigiGait Image Analysis software. In each behavioral test, the mice were trained at a rate of 15 cm/s for 7 days prior to the induction of SCI before they were tested at a rate of 9 cm/s and their images were recorded with a high-speed camera.

### QIIME test

To evaluate the Ace index of operational taxonomic unit (OTU) level, Shannon index of OTU level as well as the relative abundances of Lactobacillales, Clostridiales and Bifidobacteriales, the mice in each group were subjected to QIIME tests. In brief, paired end readings were combined by utilizing a FLASH software program. Then, quality filtering of raw tags was carried out to get high quality tags depending on the requirements in the QIIME software. In the next step, the tag information was compared to the reference information by making use of a UCHIME algorithm to identify chimera sequences, which were then eliminated to acquire effective tags. For OTU clustering as well as species annotation, all effective tags were subjected to sequence analysis by utilizing the Uparse program.

### Test of body weight, food intake, energy expenditure (EE) and respiratory quotient (RQ) in each mouse group

Total EE, which was shown as kcal/day, was actually assessed prior to as well as throughout the entire experiment by using Weir’s formula, i.e., VCO_2_ (EEVCO_2_) = 3.941 × VCO_2_ (L/min)/RQ + 1.11 × VCO_2_ (L/min) × 1440.

### Statistical analysis

All results were the average of three experiments. All data were shown as the average ± standard deviation. The correlation between BMS score and the levels of Clostridiales/Lactobacillales was analyzed by using linear regression. One-way ANOVA was utilized for comparing different groups. Statistical evaluations were done by making use of SPSS 22.0 software (SPSS, Chicago, IL). *P* < 0.05 was actually taken into consideration as statistically significant.

## RESULTS

### MOX treatment mitigated dysregulated gut bacterial composition in SCI mice

**Figure 1:**
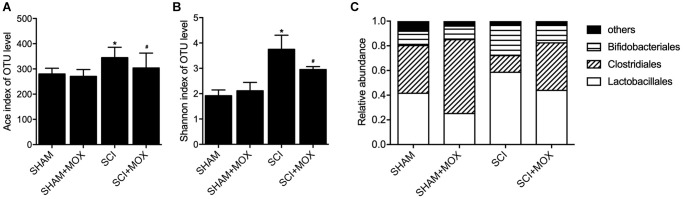
MOX treatment mitigated the dysregulation of gut bacterial composition in SCI mice (^*^P value < 0.05 vs. SHAM group; ^#^*P* value < 0.05 vs. SCI group). (**A**) Ace index in the SHAM group, SHAM+MOX group, SCI group and SCI+MOX group; (**B**) Shannon index in the SHAM group, SHAM+MOX group, SCI group and SCI+MOX group; (**C**) Relative abundance of Lactobacillales, Clostridiales and Bifidobacteriales in the SHAM group, SHAM+MOX group, SCI group and SCI+MOX group.

QIIME was used to measure the level and diversity of bacterial composition based on the OTU level in each group. Moreover, relative abundance of Lactobacillales, Clostridiales and Bifidobacteriales was also compared between different groups. As indicated by the results, the SCI group showed a significant increase in the Ace index (Figure 1A) and Shannon index (Figure 1B). Accordingly, MOX treatment significantly reduced the richness and diversity of the intestinal microbiota in SCI mice while exhibiting no effect in the SHAM group. As shown in Figure 1C and compared with the SHAM group, the induction of SCI markedly reduced the levels of Lactobacillales and Bifidobacteriales while elevating the level of Clostridiales. Accordingly, MOX treatment alleviated SCI-induced up-regulation of Clostridiales and down-regulation of Lactobacillales but exhibited no obvious effect on the level of Bifidobacteriales.

### MOX treatment mitigated the dysregulation of weight gain and metabolic profiling in SCI mice

**Figure 2:**
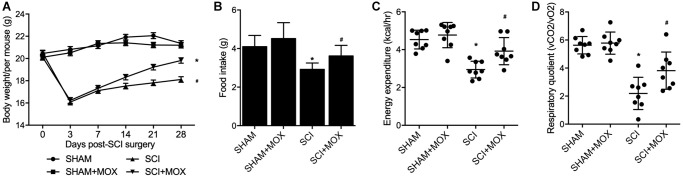
MOX treatment mitigated the dysregulation of weight gain and metabolic profiling in SCI mice (^*^P value < 0.05 vs. SHAM group; ^#^*P* value < 0.05 vs. SCI group). (**A**) Body weight during 0-28 days post-SCI surgery in the SHAM group, SHAM+MOX group, SCI group and SCI+MOX group; (**B**) Food intake in the SHAM group, SHAM+MOX group, SCI group and SCI+MOX group; (**C**) Energy expenditure in the SHAM group, SHAM+MOX group, SCI group and SCI+MOX group; (**D**) Respiratory quotient in the SHAM group, SHAM+MOX group, SCI group and SCI+MOX group.

After comparing the body weight in all mouse groups from Day 0 to Day 28 after model establishment (Figure 2A), we found that the SHAM and SHAM+MOX groups showed stable and comparable values of body weight index, but the mice in the SCI group lost body weight after surgery, and MOX treatment gradually aimed weight gain in SCI mice. In addition, metabolic parameters including food intake, EE and RQ were assessed in each group. As shown by the results, the food intake (Figure 2B), EE (Figure 2C) and RQ (Figure 2D) of SCI mice were all evidently decreased, while MOX treatment significantly mitigated the reduction in these metabolic parameters in SCI mice. Noteworthy, MOX treatment upon sham-operated mice showed no effect on the indexes, indicating that MOX treatment was only effective in SCI mice.

### MOX treatment improved locomotor recovery in SCI mice

**Figure 3:**
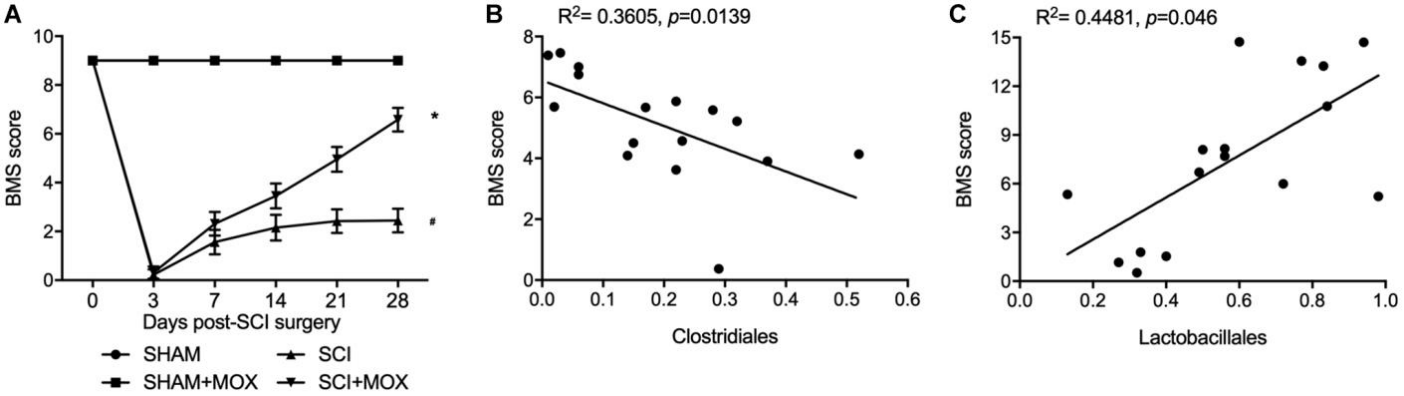
**MOX treatment improved locomotor recovery in SCI mice.** (**A**) BMS score during 0–28 days post-SCI surgery in the SHAM group, SHAM+MOX group, SCI group and SCI+MOX group (^*^P value < 0.05 vs. SHAM group; ^#^*P* value < 0.05 vs. SCI group); (**B**) Correlation analysis of BMS score and Clostridiales level in the SCI group and SCI+MOX group; (**C**) Correlation analysis of BMS score and Lactobacillales level in the SCI group and SCI+MOX group.

BMS was performed to evaluate the changes and subsequent recovery in locomotion of each group. As shown in Figure 3A, the BMS score remained unchanged in the SHAM and SHAM+MOX groups but was evidently reduced in the SCI group, and the subsequent post-SCI recovery was accompanied with a stepwise increase in the BMS score. Also, mice treated with MOX exhibited a greater improvement in locomotion activity. We also analyzed the correlation between Clostridiales/Lactobacillales and BMS score. As indicated by the results, the BMS score was negatively correlated with the level of Clostridiales (Figure 3B) and positively correlated with the level of Lactobacillales (Figure 3C), indicating the possibility of using certain bacteria to predict the functional recovery after SCI.

### MOX treatment reduced SCI-induced up-regulation of IL-17, IFN-γ, MCP-1 and IL-1β expression in SCI mice

**Figure 4:**
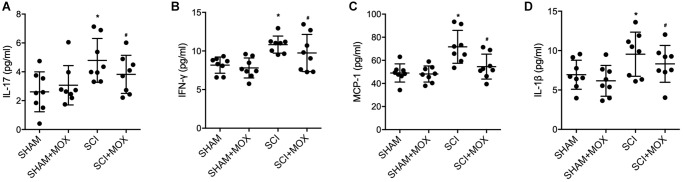
**MOX treatment reduced SCI-induced up-regulation of IL-17, IFN-γ, MCP-1 and IL-1β expression.** (**A**) Level of IL-17 in the SHAM group, SHAM+MOX group, SCI group and SCI+MOX group; (**B**) Level of IFN-γ in the SHAM group, SHAM+MOX group, SCI group and SCI+MOX group; (**C**) Level of MCP-1 in the SHAM group, SHAM+MOX group, SCI group and SCI+MOX group. (**D**) Level of IL-1β in the SHAM group, SHAM+MOX group, SCI group and SCI+MOX group.

ELISA was performed to measure the levels of IL-17, IFN-γ, MCP-1 and IL-1β in each mouse group. Compared with those in the SHAM group, the levels of IL-17 (Figure 4A), IFN-γ (Figure 4B), MCP-1 (Figure 4C) and IL-1β (Figure 4D) were increased in the SCI group, and MOX treatment reduced the expression of IL-17, IFN-γ, MCP-1 and IL-1β in the SCI+MOX group. Moreover, no evident difference was observed between the SHAM+MOX and SHAM groups.

### MOX treatment preserved colonic macrophage phenotypes in SCI mice

**Figure 5:**
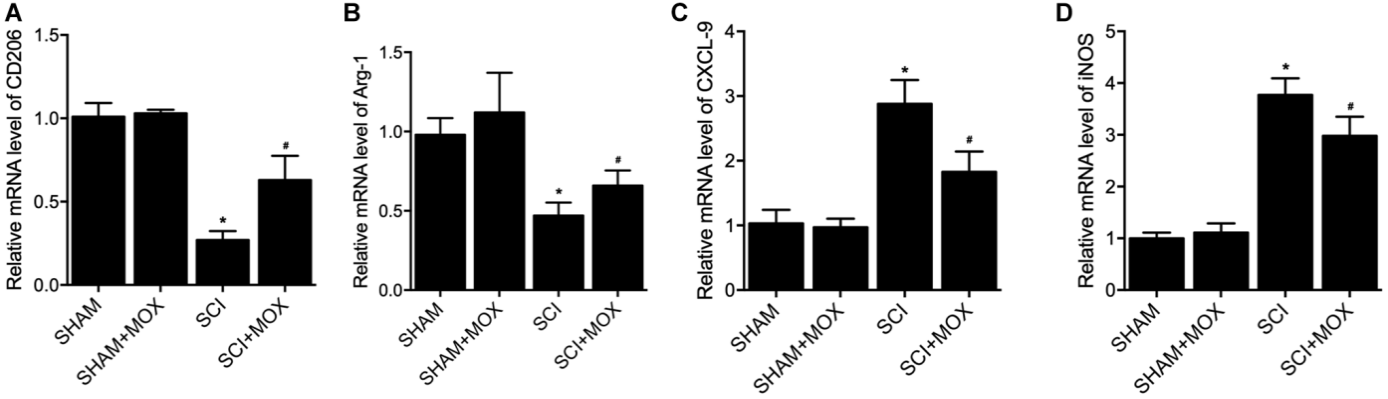
**MOX treatment preserved colonic macrophage phenotypes in SCI mice.** (**A**) Level of CO206 mRNA in the SHAM group, SHAM+MOX group, SCI group and SCI+MOX group; (**B**) Level of Arg-1 mRNA in the SHAM group, SHAM+MOX group, SCI group and SCI+MOX group; (**C**) Level of CXCL-9 mRNA in the SHAM group, SHAM+MOX group, SCI group and SCI+MOX group; (**D**) Level of iNOS mRNA in the SHAM group, SHAM+MOX group, SCI group and SCI+MOX group.

To measure macrophage polarization in each group, the expression of M1 markers including CXCL-9 and iNOS, as well as the expression of M2 markers including CD206 and Arginase-1, were observed in colonic macrophages. As shown in Figure 5, the level of M2 markers CD206 mRNA (Figure 5A) and Arg-1 mRNA (Figure 5B) were decreased in SCI mice, while the levels of M1 markers CXCL-9 mRNA (Figure 5C) and iNOS mRNA (Figure 5D) were increased in SCI mice. Furthermore, the dysregulation of these markers in SCI mice was recovered by the treatment with MOX.

### MOX treatment suppressed cell apoptosis in SCI mice

**Figure 6:**
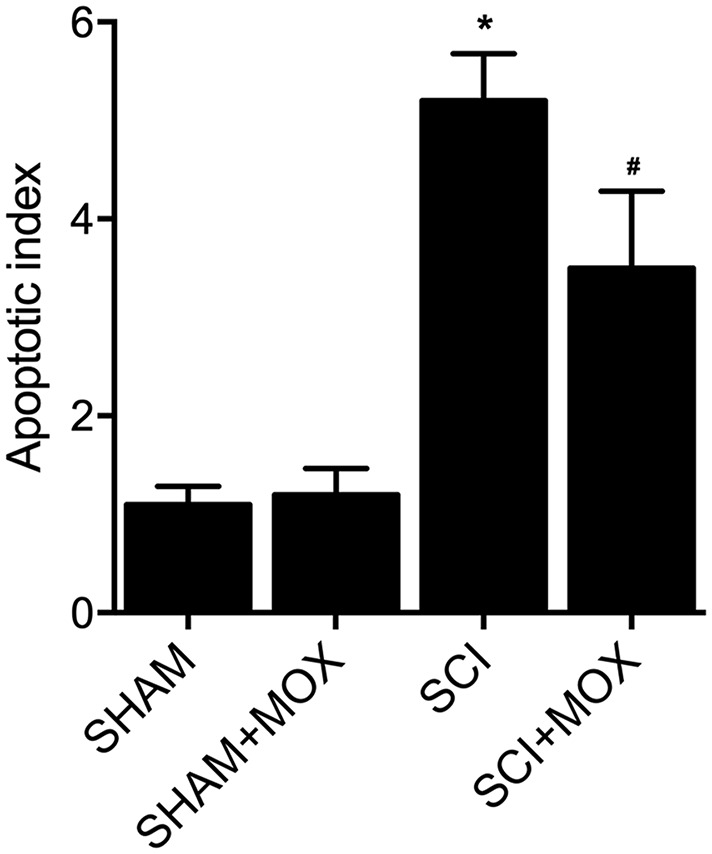
TUNEL assay validated that MOX treatment suppressed cell apoptosis in SCI mice (^*^P value < 0.05 vs. SHAM group; ^#^*P* value < 0.05 vs. SCI group).

**Figure 7:**
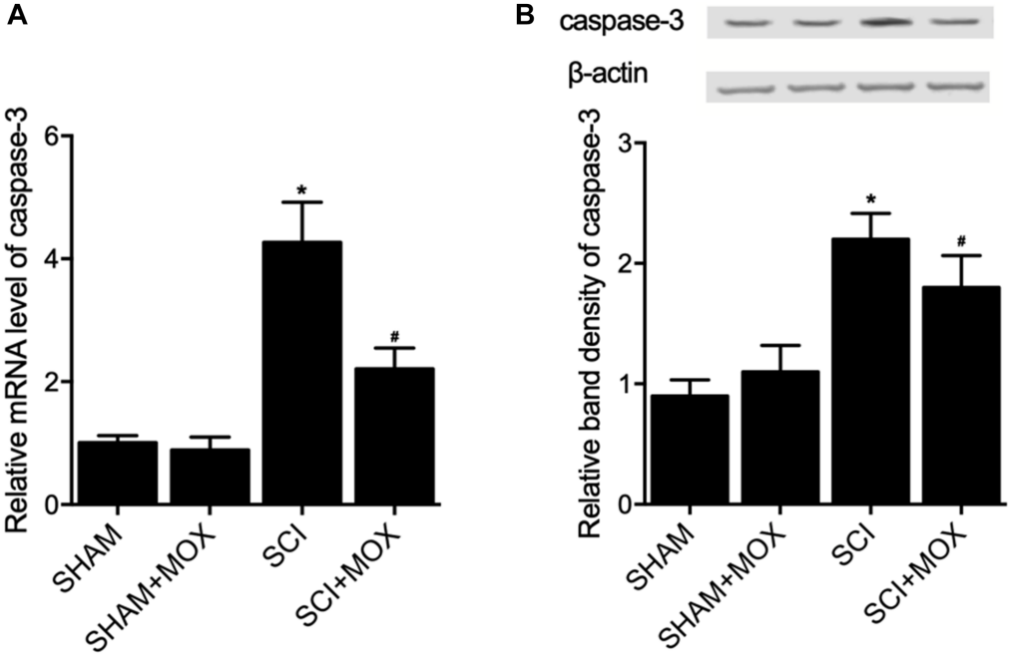
MOX treatment suppressed SCI-induced up-regulation of caspase-3 expression in SCI mice (^*^P value < 0.05 vs. SHAM group; ^#^*P* value < 0.05 vs. SCI group). (**A**) Relative mRNA level of caspase-3 in the SHAM group, SHAM+MOX group, SCI group and SCI+MOX group; (**B**) Relative band density of caspase-3 protein in the SHAM group, SHAM+MOX group, SCI group and SCI+MOX group.

TUNEL assay was utilized to measure the apoptosis of cells in spinal cord tissues. As shown in Figure 6, the apoptotic index was most evidently increased in the SCI group, while the SCI+MOX group showed an apoptotic index lower than that in the SCI group. Moreover, compared with that in the SHAM group, the expression of caspase-3 mRNA (Figure 7A) and protein (Figure 7B) was also increased after the induction of SCI, and was reduced by MOX treatment in the SCI+MOX group.

## DISCUSSION

Moxibustion was analyzed in many IBD animal models and appeared to alleviate inflammation in rodents by lowering the expression of TNF receptors [22, 23]. Moxibustion can likewise upregulate the expression of tight junction protein to reduce the apoptosis of epithelial cells [24, 25]. Furthermore, rats with DSS induced colitis showed enhanced expression of inflammatory cytokines, minimized expression of anti-inflammatory cytokines, as well as gut dysbiosis inflamed, and the effects of DSS could be alleviated by moxibustion therapy, which exerted its curative effects through regulating the microbiome as well as the immunity in intestinal tract mucus [13]. In this study, we found that the Ace index and Shannon index in the SCI group were significantly increased, while the MOX treatment significantly reduced the values of above indexes. The relative abundance of Lactobacillales and Bifidobacteriales was reduced while the relative level of Clostridiales was elevated in the SCI group, while the MOX treatment mitigated the above issues. Meanwhile, we also found that the BMS score remained unchanged between the SHAM and SHAM+MOX groups but was evidently reduced in the SCI group, while the MOX treatment greatly improved locomotion activity. Accordingly, the BMS score was negatively correlated with the level of Clostridiales and positively correlated with the level of Lactobacillales.

The impact of moxibustion might travel a long distance as a result of neural reflexes [26]. Past researches have actually uncovered that moxibustion can activate distinct cytokines as well as signaling pathways while enhancing the autophagic activity of macrophages [9, 11, 25, 27, 28]. Additionally, dysbiosis might promote the pathogenesis of post-SCI intraspinal disorders, and recent information revealed that dysbiosis after SCI triggers the reduction of butyrate-producing bacteria in the gut [29–31]. That is why regular use of probiotics containing lactic acid bacteria such as Lactobacillus as well as Bifidobacterium can actually promote post-SCI healing in mice [17, 32, 33]. At the same time, it was shown that gut Lactobacillus as well as Bifidobacterium levels were increased after SCI, suggesting the therapeutic outcome of probiotics in the SCI treatment.

Nonetheless, numerous factors can influence the curative outcome of regulating gut dysbiosis. The immune responses are important post-SCI physiological processes, and the activation of macrophages has been shown to be a necessary step in triggering such responses [34, 35]. The differing levels of the M1 as well as M2 macrophages can determine the profile of post-SCI immune responses [36]. The M1 as well as M2 macrophages may be individually modulated by cytokines released from Th2 and Th1 helper cells, respectively [37]. Microbial stimulations, including lipopolysaccharides as well as cytokines related to Th1 helper cells like interferon-γ can promote M1 polarization of macrophages, which exert pro-inflammatory, tumor resistance as well as microbicidal effects due to their ability of antigen presentation and higher yield of IL-6, IL-23 as well as IL-12, along with high level of secretion of reactive oxygen intermediates and NO [38–41]. On the other hand, Th2 cytokines like IL-4 as well as IL-13 can promote M2 polarization of macrophages, which exert anti-inflammatory, tumor promoting as well as parasite clearance effects [42]. In this study, we found that the levels of IL-17, IFN-γ, MCP-1 and IL-1β were increased in the SCI group, and MOX treatment reduced the levels of IL-17, IFN-γ, MCP-1 and IL-1β in the SCI+MOX group. Similarly, the levels of M2 markers CD206 mRNA and Arg-1 mRNA were decreased but the levels of M1 markers CXCL-9 mRNA and iNOS mRNA were increased in SCI mice, while the dysregulation of these markers was alleviated by the MOX treatment. The phenotype change of macrophages can be impacted by the changes in post-SCI neuro-inflammatory environment [43]. It was proposed that both macrophages and microglia might be polarized in reaction to various stimulations, including the presence of various cytokine clusters [44–46]. Furthermore, both IL-13 and IL-4 are anti-inflammatory and are cytokines frequently utilized for inducing alternative polarization in Raw264.7 macrophages. In addition, IL-13 and IL-4, along with their receptors IL-13R and IL-4R, are important signals required to induce above polarization [47].

## CONCLUSION

By investigating the effect of MOX on SCI mice, it can be concluded that the treatment with MOX promoted microbiota dysbiosis and macrophage polarization, while alleviating spinal cord injury via the down-regulation of inflammatory cytokines.

## Supplementary Materials

Supplementary Figure 1
