# Supplementary figures and images for: Moxibustion exhibits therapeutic effects on spinal cord injury via modulating microbiota dysbiosis and macrophage polarization
Source: Aging (Albany NY). 2022 Jul 21;14(14):5800–11. doi: 10.18632/aging.204184 (PMC9365548; doi:10.18632/aging.204184)

## SUPPLEMENTARY FIGURE

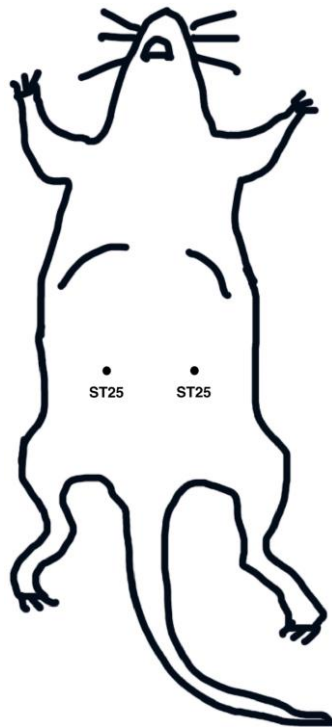

Supplementary Figure 1. Positions of ST25 acupoint.

Supplement: Supplementary Figure 1 [file aging-14-204184-s001.pdf]
